# Parathyroid cysts: experience of a rare phenomenon at a single institution

**DOI:** 10.1186/s12893-018-0340-4

**Published:** 2018-02-06

**Authors:** Peipei Xu, Xiaotian Xia, Meifang Li, Minggao Guo, Zhili Yang

**Affiliations:** 1 Department of General Surgery, Sixth People’s Hospital Affiliated Shanghai Jiao Tong University, 600 Yi-Shan Road, Shanghai, 200233 China; 2 Department of Emergency, Sixth People’s Hospital Affiliated Shanghai Jiao Tong University, 600 Yi-Shan Road, Shanghai, 200233 China

**Keywords:** Parathyroid cyst, Parathyroid hormone, Thyroidectomy, Cystectomy

## Abstract

**Background:**

Parathyroid cysts are relatively uncommon lesions and are often misdiagnosed. We evaluate our experience in the diagnosis of and therapy to correct parathyroid cystic lesions.

**Methods:**

We retrospectively reviewed a series of 32 patients with parathyroid cysts who were admitted to our department between July 2011 and November 2016. Clinical pathological features of the patients, including age, gender, location, size, ultrasonography, histopathology, surgery, and follow-up, were analyzed.

**Results:**

There were 22 female and 10 male participants with a median age of 46.7 years old (27–76 years old). Only two cysts were found in the superior mediastinum. The rest were located under the lower pole of the thyroid. All of the patients underwent ultrasonography scans and serum parathyroid hormone (PTH) assays. Three patients had elevated serum PTH levels, and they were further scanned with Tc99m sestamibi as functional cysts. In 29 cases of nonfunctional cysts, 3 cases were preoperatively diagnosed by cystic aspiration with PTH detection. The rest were diagnosed by postoperative immunopathology. All of the patients underwent cystectomy, and 24 patients also underwent thyroidectomy. There was a significant difference in cyst diameter size between the cystectomy alone and cystectomy with thyroidectomy groups (4.0 ± 2.0 vs 1.5 ± 1.0 cm; *p* < 0.05). No participant experienced recurrence during the median 36 months of follow-up.

**Conclusions:**

Cystic lesions located under the lower pole of the thyroid gland should be considered to have originated at the parathyroid gland. Cystic aspiration with PTH detection or postoperative immunopathology can lead to a definitive diagnosis. Cystectomy is still a commonly used and effective treatment.

## Background

Parathyroid cysts (PCs) are a rare entity, and only 300 cases have been reported worldwide [1]. Clinically, they are defined as either functional or non-functional based on the presence or absence of hyperparathyroidism [2]. Most PCs are non-functional and commonly present as asymptomatic nodular cervical lesions [3]. They are often mistaken for other neck masses, such as thyroid cysts. There are three options for treatment of PC: aspiration, percutaneous injection of sclerosing agents, and surgical resection [4, 5]. At present, the diagnosis and treatment of PC is limited to a few small case reports. We reviewed 32 cases of PC in our institution, summarized their clinical features, and highlighted the diagnosis and treatment procedures.

## Methods

We retrospectively reviewed a series of cases of PCs in patients who had been admitted to our department between July 2011 and November 2016. Age, gender, cyst location and size, laboratory and imaging results, treatment, and pathology were recorded. All of the patients underwent a histological examination for their final diagnosis. In this cohort, the indications for thyroidectomy include nodules with suspected malignant sonographic features, nodules confirmed as malignant by fine needle aspiration cytology (FNAC), and benign nodules that are larger than 3 cm in diameter.

The surgical procedure involves a cystectomy alone or combined with thyroidectomy through a middle-access incision under the effect of general anesthesia. Briefly, the patient is positioned with the neck extended, and a 3- to 5-cm collar-type incision is made; two skin flaps are created by dissecting them away from the strap muscles, and the thyroid capsule is approached by splitting the strap muscles along the midline. According to the preoperative location and by exposing the lower pole of the thyroid and dissecting the superficial fascia, the PC will be found. To avoid injury of the recurrent laryngeal nerve, the surrounding fascia was dissociated along the cyst wall, the vessels were cut with an ultrasonic scalpel, and the cyst was removed and sent off for pathological examination. Two mediastinal PCs were removed using videothoracoscopy. The corresponding conventional thyroidectomy was performed for the concomitant thyroid diseases (e.g., malignant or benign nodules). Intraoperative serum PTH (IOPTH) was measured 10 min after the excision of 3 functional PCs.

Serum calcium was measured in the postoperative 24 h for all of the cases and at 48 h, 72 h and 1 w for FPC cases. Calcium was administered intravenously in postoperative hypocalcemia functional parathyroid cyst (FPC) cases, and 2–3 days later, it was changed to oral calcium for 3 months. For nonfunctional parathyroid cysts (NFPCs), it is generally not routine to use calcium supplementation after surgery. All of the patients with PC underwent reexamination of the neck ultrasound, blood PTH and serum calcium once a year except for the FPC patients, whose blood PTH and serum calcium were examined in the postoperative 3 month period. All of the enrolled patients were followed up by making an appointment and dialing the reserved phone number. The diameters of all PCs are reported as the means ± standard error and were compared between the cystectomy alone group and the cystectomy combined with thyroidectomy group using Student’s *t* test. *P*-values < .05 were regarded as statistically significant. Statistical analysis was performed with SPSS 20.0 (SPSS Inc., Chicago, Illinois).

This retrospective study was endorsed by our hospital ethics committee, and all patients signed informed consent forms.

## Results

We identified 32 patients (22 female and 10 male) with a diagnosis of PC. Their median age was 46.7 years (27–76 years). A total of 30 PCs were located under the lower pole of the thyroid gland, and 2 were in the superior mediastinum; their diameters ranged from 1 to 6 cm, and the diameters of only 6 cysts were more than 2.5 cm. All of the patients were asymptomatic, and only 5 complained of an anterior cervical mass (Table 1).

**Table 1:** Clinical characteristics of PC patients in our institution

Parameter	Value
Gender (female/male) (No.)	22/10
Age (<40/>40 years)	12/20
Size (diameter < 2.5/>2.5 cm)	26/6
Serum PTH (high/normal pg/ml)	3/29
Location (superior mediastinum/under the inferior pole of the thyroid gland) (No.)	2/30
Neck ultrasonigrapy (yes/no) (No.)	32/0
Cyst aspiration PTH measure (yes/no) (No.)	3/29
Surgery (simple cyst resection/thyroidectomy and cystomy) (No.)	8/24
Flollow-up (recurrence/no recurrence) (No.)	0/32

**Fig. 1:**
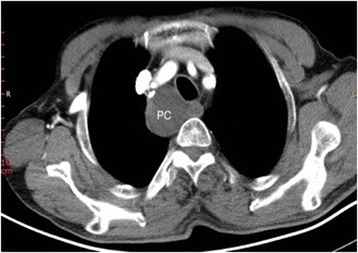
Representative case with enhanced computer tomography scan showing a huge cyst in the superior mediastinum. Cystic lesion (4×3 cm); PC, parathyroid cyst

**Fig. 2:**
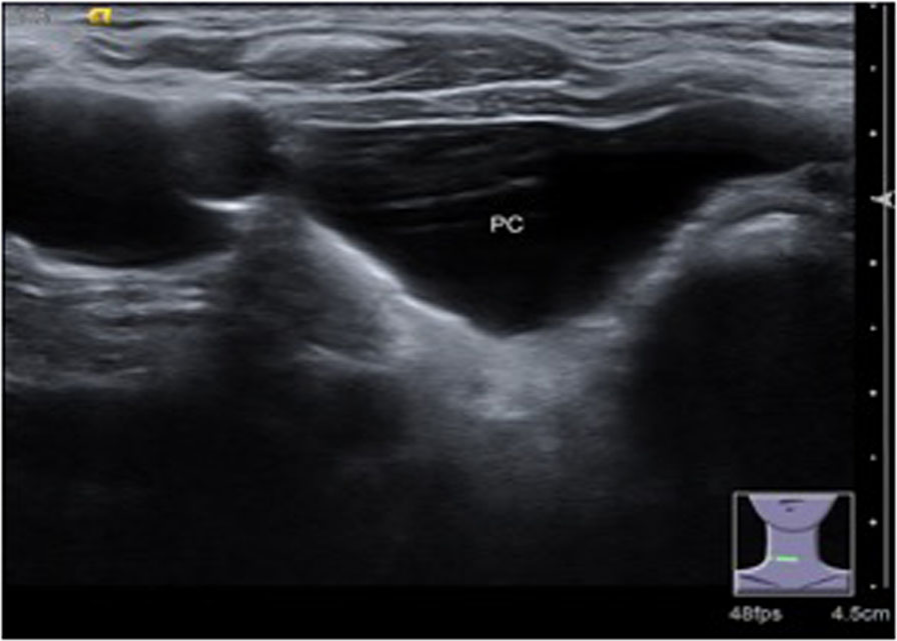
Representative case with PC ultrasonography scans. Cystic lesion (2.5×2×0.8 cm) in right lobe of thyroid gland. PC, parathyroid cyst

**Fig. 3:**
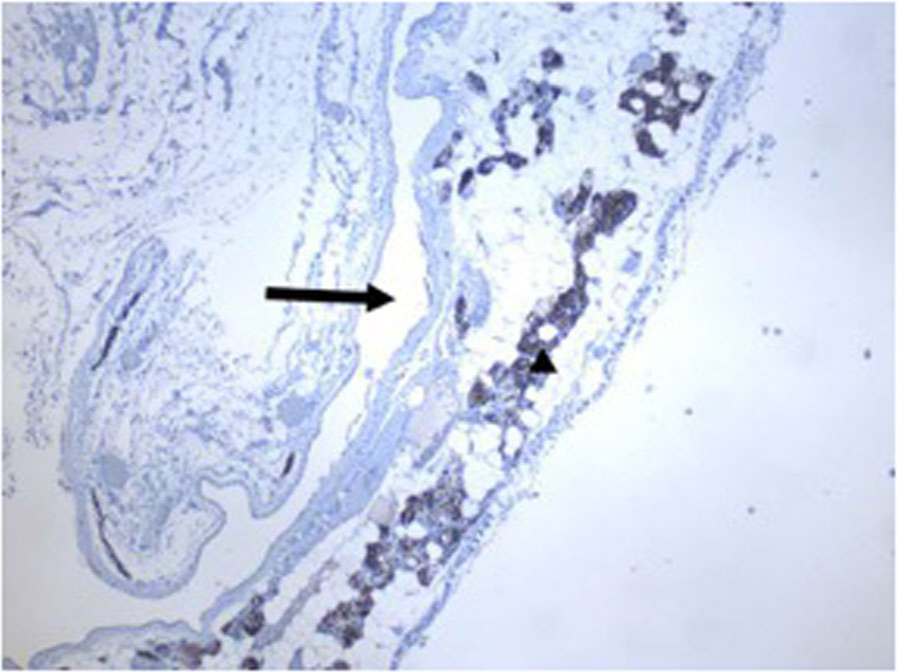
Representative case with immunopathology of parathyroid cysts. Cytoplasmically stained blown cells with antibody against PTH are parathyroid cells (arrow); cyst lumen (black asterisk) (40×)

Two cysts were found incidentally in the superior mediastinum by enhanced computer tomography. Each was a large cyst that was later confirmed to be a PC by postoperative immunopathology (Fig. 1). Ultrasonography scans and PTH assays in the serum were performed preoperatively in all patients. Ultrasonography scans reported that the area under the lower pole of the thyroid was free of echoes (Fig. 2). Only three patients with elevated serum PTH levels (350 pg/ml, 324 pg/ml, and 224 pg/ml) were further examined with a Tc99m sestamibi scan, which presented a delayed imaging of the parathyroid. Cystic aspiration with PTH detection (540 pg/ml, 326 pg/ml, and 370 pg/ml) was conducted in 3 of the 29 patients with normal serum PTH levels, and the diagnosis of PC was confirmed by postoperative immunopathology (Fig. 3).

**Fig. 4:**
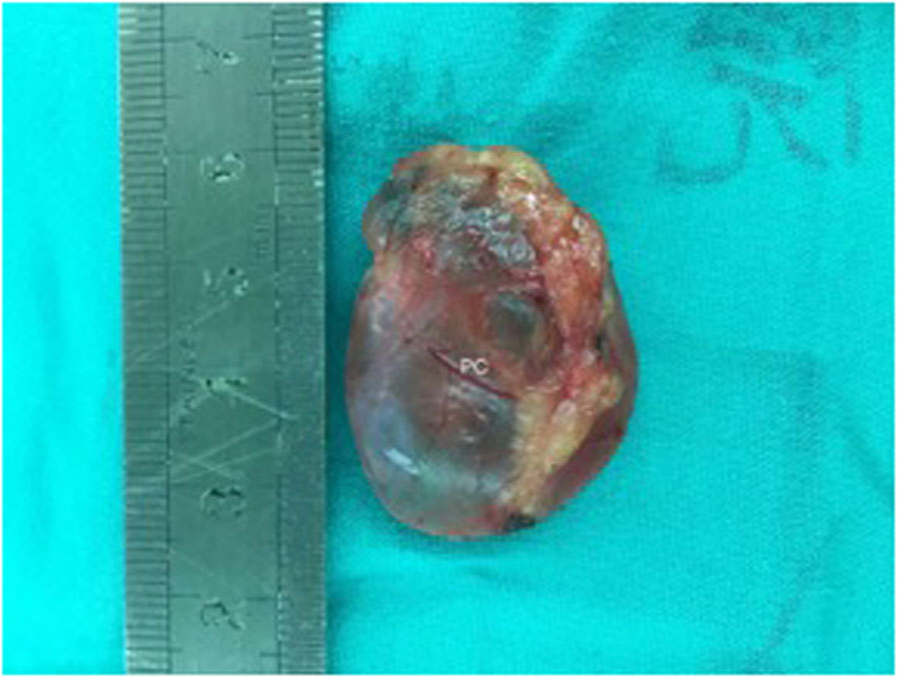
Representative parathyroid cysts as seen intra-operatively. PC, parathyroid cyst

All of the patients underwent cystectomy (Fig. 4). Of these patients, 8, including 3 patients with high serum PTH levels, underwent cystectomy alone, and the remaining patients also underwent thyroidectomy for other thyroid-related conditions. The median hospital stay was 5 days (range: 3–8 days). There was a significant difference in cyst diameter size between the cystectomy alone and cystectomy with the thyroidectomy groups (4.0 ± 2.0 vs 1.5 ± 1.0 cm; *p* < 0.05). Only 2 cases of FPCs had transient hypocalcemia in the postoperative 24 h and were remitted in the postoperative 1 w period. There were no other complications in any of the surgical cases. No participant suffered recurrence during the median 36 months of follow-up (12–60 months), as indicated by ultrasonography scans and their serum calcium and PTH levels.

## Discussion

PC is a rare entity in clinical practice, accounting for 0.8–3.41% of all parathyroid lesions and affecting 0.075% of the unselected population [6]. The first report of surgical resection of PC was made in 1905 by Goris [7]. In 1953, Crile diagnosed PC for the first time by FNA [8]. Currently, only approximately 300 cases have been reported worldwide.

In this series of cases, PCs were more common in women, and patients had a median age of 46.7 years. Almost all of the lesions are located under the lower pole of the thyroid, and the diameters ranged from 1 cm to 6 cm. Based on these demographic characteristics, the pathogenesis of PCs may be related to the following factors. This cyst may have been caused by a vestigial remnant of the third or fourth branch or the persistence of the Kürsteiner canals [3]. The PC may have been formed from infarct and degeneration of paralytic adenoma or by the development of multiple microcapsules in normal parathyroid tissue [9]. There are no reports on the exact mechanism of the evolution of parathyroid cysts with hyperplasia and adenoma formation.

Ultrasound is faster, cheaper, and more widely available than other imaging techniques, and it is highly accurate in detecting neck masses and evaluating the local extent of disease [10]. Although it cannot distinguish between PC and other cervical diseases, ultrasonography may reveal a nonspecific cystic structure and establish its proximity to the thyroid gland [4, 11]. In this review, ultrasonographic scans were performed for all of the patients. Here, 30 were positioned under the lower pole of the thyroid, and 2 were ectopic. In this way, the diagnosis of a suspected PC can be established by ultrasonography and based on the location of cystic masses, which were under the lower pole of the thyroid. As a complement, a CT of the head and neck may indicate the cystic components of these lesions and help establish the relationship to adjacent tissues [12]. This may be particularly helpful in the presence of substernal extension or compressive symptoms. Although most of the PCs are accompanied with thyroid nodules in this cohort, it may be difficult to speculate that the occurrence of PC is associated with thyroid disease. To date, there have been eight intrathyroidal PC cases reported in the literature [13]. No other reports of possible relationships between PC and thyroid disease have been described.

PCs can be classified as functional (FPC) or nonfunctional (NFPC) according to the presence or absence of hyperparathyroidism. In our series of cases, 90% were NFPCs (29/32), and 10% were FPCs (3/32), which suggested that non-functional cysts are more common. NFPCs generally manifested as asymptomatic masses, which are hard to diagnose and often misdiagnosed. Among our 29 NFPCs, 26 were mistaken for other cervical diseases such as thyroid cysts, thyroglossal duct cysts, bronchial cleft cysts, and even mediastinal tumors. The remaining 3 cysts were diagnosed because of elevated PTH levels of cyst aspiration. PCs located in the mediastinum are even rarer and easier to misdiagnose as mediastinum tumors [14]. In our report, 2 cysts found incidentally in the superior mediastinum by enhanced computer tomography were misdiagnosed as tumors and confirmed by postoperative immunopathology.

The PTH assay for cystic aspiration is an effective method of diagnosing PCs, especially NFPCs [15]. There were 3 NFPCs diagnosed by cystic aspiration and elevated intact parathyroid hormone (iPTH) levels. The diagnosis was confirmed with postoperative immunopathology in this series of cases. It has been reported that the tissue synthesizes large amounts of PTH with degenerated carboxy-terminal parathyroid hormone (C-PTH), which renders it inactive in the NFPC [16]. Current laboratory practice is to perform an iPTH assay, which may underestimate the PTH levels of cyst fluid. It is therefore recommended that C-PTH in cystic fluid should be measured instead of iPTH [17]. Even so, the presence of any iPTH in the cystic fluid, regardless of the level, can suggest a cyst of parathyroid origin. For this reason, as we reported in the present cases, iPTH testing may not affect the diagnosis of PC. High-performance liquid chromatography may be a suitable means of accurately measuring the concentration of PTH in fluid taken from cysts.

A high level of serum PTH is one of the most important parameters in the diagnosis of functional parathyroid cysts. Meanwhile, ultrasound and 99 m Tc-MIBI parathyroid imaging can be used for positional and functional diagnosis [18]. The results have shown that serum PTH was significantly higher than normal, and ultrasound and 99 m Tc-MIBI were used to precisely determine the location of the lesion within the parathyroid in our 3 cases of FPC. In this way, if the serum PTH is significantly higher than normal, the cystic lesions under the lower pole of the thyroid can be diagnosed easily using FPC. The possibility of rapidly developing adenocarcinoma should also be considered in FPC patients [19–22].

In this cohort, we realized from the following three aspects that the diagnosis of NFPC can be improved. First, from the imaging features of the lesions, almost all were free echo (ultrasound) or low density (CT), and there were no parenchymal tissue features. Second, most of the lesions are located under the lower pole of the thyroid gland. Third, in the laboratory examination, iPTH was detected in the ultrasound-guided puncture solution.

**Fig. 5:**
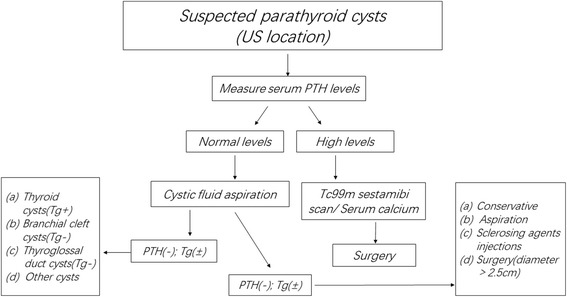
Process for evaluation and management of patients with suspected parathyroid cyst. PTH, parathyroid hormone; Tg, thyroglubin

The best treatment for FPC is surgical removal of the lesion, but for NFPC, surgical treatment is optional. There are three options for the treatment of NFPC including simple aspiration, percutaneous sclerosing agent injections and surgical resection [4, 5]. Ultrasound-guided aspiration can be used alone as an initial treatment for symptomatic NFPCs, and it can immediately resolve compression symptoms. However, existing literature has shown that aspiration alone is common in cases of small NFPCs with diameters less than 2.5 cm. Even so, there are still reports of recurrence during follow-up. Some cases even repeatedly relapse after multiple aspirations and are finally treated by surgical resection or ethanol ablation [23–26]. These reports suggest that the larger the cyst is, the more likely it is to relapse after the procedure. Sclerosing agent injections are not recommended because they can flow out from the capsule and cause serious complications such as fiber degeneration or damage to the recurrent laryngeal nerve, resulting in vocal cord paralysis [26, 27]. It has been suggested that the optimal treatment for symptomatic NFPCs is surgical resection [1, 28]. In the present report, 5 patients with NFPC (diameter greater than 2.5 cm) underwent cyst resection alone. Another 24 patients underwent thyroidectomy with parathyroid cystomy (diameter under 2.5 cm) due to a thyroid-related disease without any postoperative complications, and there was no recurrence during the median 36 months of follow-up. Our experience has shown that surgical resection is an effective and safe treatment for NFPCs with diameters greater than 2.5 cm. Figure 5 shows a synthesis of our cumulative experience with previously reported elegant data. We propose an algorithm to guide the diagnosis and management of PC.

## Conclusion

Cystic lesions located under the lower pole of the thyroid gland should be considered to have originated at the parathyroid gland. Cystic aspiration with PTH detection or postoperative immunopathology can be definitively diagnosed. Resection of the cyst is still a common and effective treatment.

## Abbreviations

C-PTH: Carboxy-terminal parathyroid hormone
CT: Computed tomography
FNAC: Fine needle aspiration cytology
FPC: Functional parathyroid cyst
IOPTH: Intraoperative serum parathyroid hormone
iPTH: Intact parathyroid hormone
NFPC: Nonfunctional parathyroid cyst
PCs: Parathyroid cysts
PTH: Parathyroid hormone
